# Single Cell Analysis of Yeast Replicative Aging Using a New Generation of Microfluidic Device

**DOI:** 10.1371/journal.pone.0048275

**Published:** 2012-11-08

**Authors:** Yi Zhang, Chunxiong Luo, Ke Zou, Zhengwei Xie, Onn Brandman, Qi Ouyang, Hao Li

**Affiliations:** 1 The State Key Laboratory for Artificial Microstructures and Mesoscopic Physics, Center for Quantitative Biology, and School of Physics, Peking University, China; 2 Department of Biochemistry and Biophysics and California Institute for Quantitative Biosciences, University of California San Francisco, San Francisco, California, United States of America; 3 Department of Cellular and Molecular Pharmacology, University of California San Francisco, San Francisco, California, United States of America; 4 Department of Physics, Peking-Tsinghua Center for Life Sciences, Peking University, Beijing, China; University of Kent, United Kingdom

## Abstract

A major limitation to yeast aging study has been the inability to track mother cells and observe molecular markers during the aging process. The traditional lifespan assay relies on manual micro-manipulation to remove daughter cells from the mother, which is laborious, time consuming, and does not allow long term tracking with high resolution microscopy. Recently, we have developed a microfluidic system capable of retaining mother cells in the microfluidic chambers while removing daughter cells automatically, making it possible to observe fluorescent reporters in single cells throughout their lifespan. Here we report the development of a new generation of microfluidic device that overcomes several limitations of the previous system, making it easier to fabricate and operate, and allowing functions not possible with the previous design. The basic unit of the device consists of microfluidic channels with pensile columns that can physically trap the mother cells while allowing the removal of daughter cells automatically by the flow of the fresh media. The whole microfluidic device contains multiple independent units operating in parallel, allowing simultaneous analysis of multiple strains. Using this system, we have reproduced the lifespan curves for the known long and short-lived mutants, demonstrating the power of the device for automated lifespan measurement. Following fluorescent reporters in single mother cells throughout their lifespan, we discovered a surprising change of expression of the translation elongation factor TEF2 during aging, suggesting altered translational control in aged mother cells. Utilizing the capability of the new device to trap mother-daughter pairs, we analyzed mother-daughter inheritance and found age dependent asymmetric partitioning of a general stress response reporter between mother and daughter cells.

## Introduction

The budding yeast Saccharomyces Cerevisiae, a simple single-celled organism, has served as an important model for aging research. In the past few decades, genetic studies have identified a number of conserved pathways that regulate lifespan across species [1]. Such studies have helped establish the modern field of the molecular genetics of aging. Yeast is also one of the favorable model organisms for studying aging, due to its short lifespan and the relative ease of genetic manipulation. In addition, recent functional genomic studies have revealed a large number of regulatory interactions from which a global gene regulatory network is beginning to emerge. Knowledge of such a network makes it possible to study aging from a systems perspective.

**Figure 1:**
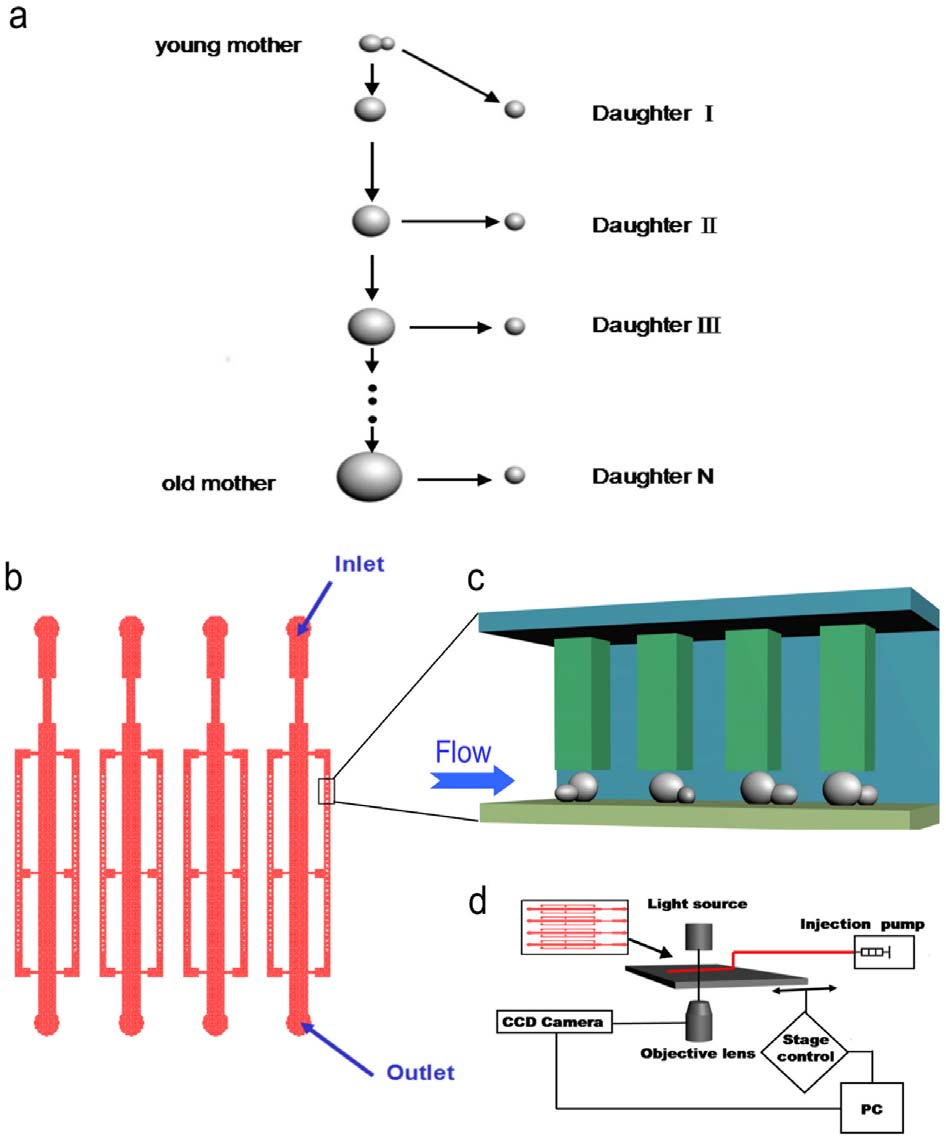
The design of the microfluidic system for yeast aging analysis (a) a schematic of the replicative aging of a yeast cell. The replicative lifespan (RLS) of a mother cell is defined as the number of daughter cells it produced throughout its life. (b) The design of the microfluidic chip. The whole device consists of multiple independent modules operating in parallel. Each module is made of one main channel connected with two side channels with arrays of pensile columns. An additional bridge is added in the middle to connect the main channel with the two side channels. (c) Mother cells are physically trapped in the gap between the soft PDMS pensile column and the glass surface. The typical size of the gap is 4∼5 µm. Since the daughter cells are generally smaller than the mother cells, they are removed automatically by the media flow. (d) The experimental setup. Cells are loaded into the microfluidic chip, which is connected to automatic pumps. Continuous images are taken by a time-lapsed microscope at multiple positions each containing one or a few pensile columns.

The phenomenon of yeast replicative aging was discovered about half a century ago, when Mortimer and Johnston reported that single yeast cells have finite replicative lifespan (RLS), defined as the number of daughter cells a mother cell can produce throughout its life [2] (Fig. 1a). The original lifespan assay, as devised by Mortimer and Johnston, was to grow virgin mother cells on a agar plate and remove daughter cells from their mothers by micro-dissection using a micromanipulator (a microscope with a dissection needle and a movable stage). Removing daughter cells is absolutely necessary in order to track the lifespan of mother cells. Without the removal of the daughter cells, the cell population will quickly expand to a big clone in less than 10 generations, which is much shorter than the typical life span of a mother cell (25 generations on average).

50 years after the initial discovery by Mortimer and Johnston, manual micro-dissection remains the canonical method for yeast lifespan analysis. This has become a major bottleneck limiting the progress of the field. The traditional method is laborious and time-consuming, make it very difficult to perform large-scale screening for genetic mutations that extend the lifespan. More importantly, with the traditional assay, it is almost impossible to follow molecular markers throughout the lifespan of the mother cells. This pose a great challenge to phenotyping aging in single cells at the molecular level.

Due to its technological importance, several groups attempted to develop methods for retaining mother cells while removing daughter cells automatically [3], [4]. For example, exploring mother/daughter size difference (mother cell is in general larger than its daughters), a microfluidic device was developed that confines mother cells in micro-jails with open gates for daughter cells to escape [4]. Daughter cells can then be separated by the flow. However, such device only works for the first few generations. As the size of mother and daughter grows with age, the daughter cells eventually jam the gates.

Recently we have developed a microfluidic system that is capable of retaining mother cells in microfluidic chambers while removing daughter cells automatically throughout the lifespan of the mother cells [5]. To achieve stability, we explored two properties of budding yeast cell division: 1) usually the size of the mother cell is bigger than that of the daughter; 2) the cell wall of the daughter comes from de novo synthesis at the budding site of the mother [6]–[8], so that if the surface of the mother cell is labeled, the daughter would not inherit the label. In the device, mother cells are trapped by a combination of geometric confinement (the height of the chamber is comparable to the size of mother cells) and adhesion between biotin labeled mother cell surface and BSA-Avidin modified glass. Although effective, the requirement for surface labeling and glass modification makes the device fabrication and operation more demanding. We found that geometric confinement by itself alone is not stable and is sensitive to the height of the chamber: if it is too high, the mother cells will not be stably trapped; if it is low enough to stably trap mother cells, there is a certain probability that daughter cells will be trapped and jam the device.

Here we report the development of a new generation of microfluidic device for yeast aging study that eliminates the requirement for surface labeling (a similar device has been developed recently by Lee et al [9], see Note Added) and can be used to study problems not possible with the first generation device. Such system allows us to simplify the experimental procedure and to achieve higher success rate. The elimination of surface labeling makes it possible to generalize the study to species other than budding yeast, as the surface labeling is based on differential partitioning of the cell wall – a specific property of budding yeast. Furthermore, this design allows trapping of the daughter cell of a trapped mother cell and the probability of trapping daughter cells can be adjusted by changing the cross section of the columns. This allows us to analyze mother/daughter asymmetry as a function of age, which is not possible with the first generation device since trapping daughter cells will eventually lead to jamming of the whole device.

Using this device, we have reproduced the lifespan curves of long and short-lived mutants, discovered a surprising change of the expression of a translation elongation factor (generally believed to be constitutively expressed) in single mother cells during aging, and analyzed asymmetric partitioning of a stress response reporter between mother and daughter cells as a function of mother age.

## Results

### Design of the microfluidic device

To achieve differential retention of mother cells by geometric confinement only, we designed arrays of pensile columns inside the microfluidic channels that can physically trap mother cells in the gap between the column and the glass surface, while allowing the removal of daughter cells automatically by the flowing media (Fig. 1c). There is a key difference between the new and the previous design [5]: in the new device, the cells are trapped only underneath the columns thus the size of the micro-colonies is limited by the size of the columns – cells growing out of the gap are flushed away. In this way we solved the possible problem of jamming in the first generation device in which cells are trapped by the whole microfluidic chamber.

Fig. 1b shows a schematic of the design of the device which contains multiple independent functional modules that can operate in parallel. Multiple strains can be analyzed simultaneously on one chip by connecting different modules to different pumps. Each module consists of one main channel connected with two side channels where an arrays of ∼100 pensile columns are located. An additional bridge is added in the middle to connect the main channel and the side channels, to avoid the possibility that the whole side channel is blocked by air bubbles. We have experimented with different sizes of the gap between the bottom of the column and the glass surface, and found that 4 µm (which is comparable to the size of yeast cells) is appropriate for trapping mother cells while allowing the removal of daughter cells. We have also tested different sizes of the columns, ranging from 20 µm by 20 µm to 80 µm by 80 µm, and found 40 µm by 40 µm is an optimal parameter. This design ensures that the trapped cells stay stably underneath the pensile columns and thus allows us to track mother cells by both bright field and fluorescent imaging throughout their entire lifespan with a time-lapsed microscope (Fig. 1d, Supplementary Movie S1).

### Loading and Tracking the cells

When loading the yeast cells into the microfluidic device, we apply a sudden injection of the yeast cell suspension with a high velocity [10](> = 1000 µl/h).The elastic microfluidic channels will swell under the high pressure induced by the flowing media, so that the gap between the columns and the glass surface will expand. Under this condition some yeast cells will be loaded into the gap. After a short pulse of high-speed injection, we lower the flow speed to release the pressure, the pensile columns resume to their original shape, and consequently the cells are trapped underneath the columns (Fig. 1c). After the initial loading, a continuous flow of fresh media is supplied by a programmable pump. The flow of fresh media keeps the micro-environment of cells constant and provides a shear force to separate and remove the daughter cells from the trapped mother cells. As the small colonies grow underneath the columns, progenies get pushed out of the gap and flushed away by the flowing media. In a typical experiment, each column can trap 1∼2 mother cells stably throughout their lifespan, thus in each functional module, we can track ∼100 mother cells, which is sufficient to provide good statistics on lifespan and other molecular markers we observe.

### Measuring the replicative lifespan using the microfluidic device

In the traditional replicative lifespan assay, an experiment always begins with virgin daughter cells that never bud before [11], [12]. With our cell loading protocol, the virgin daughter cells and mother cells with different ages can be trapped by the pensile columns initially. Theoretically more than half of the yeast cells in an exponential growth culture should be virgin daughters and first generation mothers [13]. However, trapping may create potential bias towards larger (thus older) cells. We therefore calibrated the age distribution of the cells trapped by the pensile columns after the initial cell loading.

**Figure 2:**
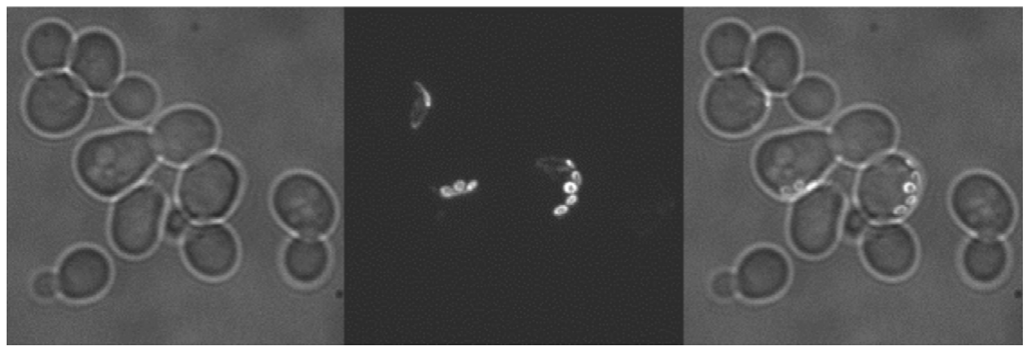
Calibration of the age distribution of the initially loaded cells. The age of the cells initially loaded into the microfluidic chip is measured by the number of bud scars as indicated by the WGA staining. On the left is a bright field image of WGA stained cells trapped by a pensile column. In the middle is the corresponding 3-D fluorescent image, reconstructed from the Z-stacks. The merged image is shown on the right.

We first stained the cells under exponential growth condition with wheat germ agglutinin (WGA) which binds specifically to chitin, the main component of the bud scar of Saccharomyces Cerevisiae [14]. WGA is coupled to FITC for visualization, this allows us to take both bright field and fluorescent images under a spinning disc confocal microscope to reconstruct a 3-D image of the cells and to count the bud scars of the cell (Fig. 2). The number of bud scars on a cell indicates the number of daughter cells it has already given birth to. We found that the average number of bud scars of the cells initially loaded underneath the pensile columns are less than 2 for pensile columns of four different sizes (Table 1). Overall the distribution of the bud scar number for initially loaded cells is very similar to that of the cells in exponentially growing cultures (Fig. S1a, b, Supplementary Table S1), thus our cell loading protocol does not create a substantial bias towards old cells. This calibration also indicates that the mean lifespan measured by the microfluidic device is about 2 generations shorter than that from the plate assay.

**Table 1:** Mean bud scars of the cells on the glass slide and under the pensile columns (Col) with different sizes.

Position	Slide	40 µm Col	50 µm Col	60 µm Col	80 µm Col
Mean Bud Scars	0.9	1.8	1.7	1.2	1.7

The number of cells tested: N_Slide = 99, N_40 µm Col = 65, N_50 µm Col = 87, N_60 µm Col = 68, N_80 µm Col = 60.

**Figure 3:**
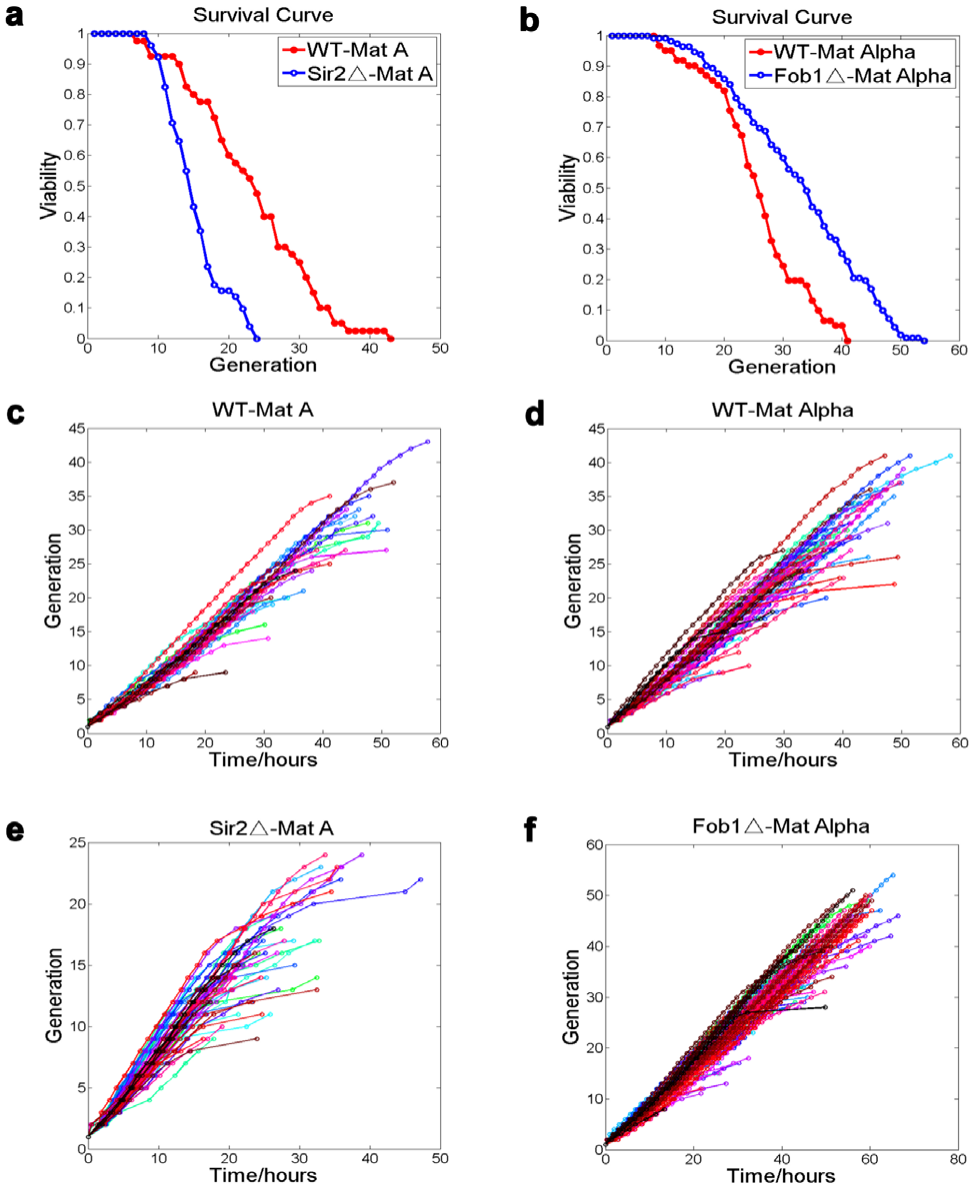
Replicative lifespan and cell division dynamics of the wild type and mutant cells measured by using the microfluidic device. (a) to (b) Survival curves for the *sir2* and *fob1* deletion strains show that they are short and long lived, respectively, compared to the wild type control (P_values from the rank-sum test: *WT* vs. *sir2Δ* = 8.1×10^−7^, *WT* vs *fob1Δ* = 2.6×10^−5^). Mean lifespan: *WT*_*MAT* ***a*** = 23.6; *sir2Δ*_*MAT* ***a*** = 15.4; *WT_MATα* = 26.0; *fob1Δ_MATα* = 33.1. The number of cells used to generate the survival curves: *WT_MAT* ***a*** = 40; *sir2Δ_MAT* ***a*** = 51; *WT_MATα* = 61; *fob1Δ_MATα* = 112. (c) to (f): cell division dynamics in single cells for the four different strains. Time at which a new bud appears on a mother cell vs. the generation (the number of daughters produced) of the mother is plotted. Data points are represented by open circles, and each colored line connects points for one single cell.

To demonstrate that the microfluidic device can be used to measure lifespan in a semi-automated fashion, we tested whether we can reproduce the lifespan data from the traditional replicative lifespan assay based on micro-dissection. We chose two genes that are well known to regulate lifespan: Sir2 and Fob1, the deletion of which shortens or extends lifespan respectively [15],[16]. We load the cells to the microfluidic chip and track the mother cells continuously (once every 10 minutes) throughout their lifespan by time-lapsed microscopy. We then count the number of daughters produced by each mother cells. Consistent with previous studies using the traditional lifespan assay, we found Sir2 deletion strain showed a decreased lifespan compared with that of the wild-type strain, while the Fob1 deletion strain showed a significantly lifespan extension (Fig. 3a, b). In addition, we found that the lifespans of the wild type cells we measured agree quantitatively with that from the traditional assay after the small calibration is made [17].

### Tracking cell division dynamics throughout the lifespan of the cells

In addition to measuring the lifespan of cells, the continuous tracking of mother cells in the microfluidic device also makes it possible to study cell division dynamics of single cells throughout their lifespan with high temporal resolution (∼10 min). This is very difficult if not impossible with the traditional approach. For example, we can record the time at which a new bud appears from a mother cell and analyze the time between two successive budding events (the budding time interval) as the function of the age of the mother (Fig. 3c–f). Consistent with the previous report, we found generally that the budding time interval and its variation increases dramatically during the last few cell divisions (Fig. S2a, b). Interestingly, we found that the budding time interval of the mother cells early in their life (around 6^th^ or 7^th^ budding events) negatively correlates with their lifespan (Fig. S2c–e) in the wild type and sir2 deletion strains, but no such correlation was found in the long-lived fob1 deletion mutant.

### Monitoring molecular markers in single cells throughout their lifespan

One of the most important applications of the microfluidic device is to track molecular markers in single cells throughout their lifespan. With this technology, we can attempt a comprehensive description of the phenotype of aging at the molecular level. Such description is essential for developing a basic understanding of the mechanistic events that drive aging and the mechanisms by which different mutants extend lifespan.

**Figure 4:**
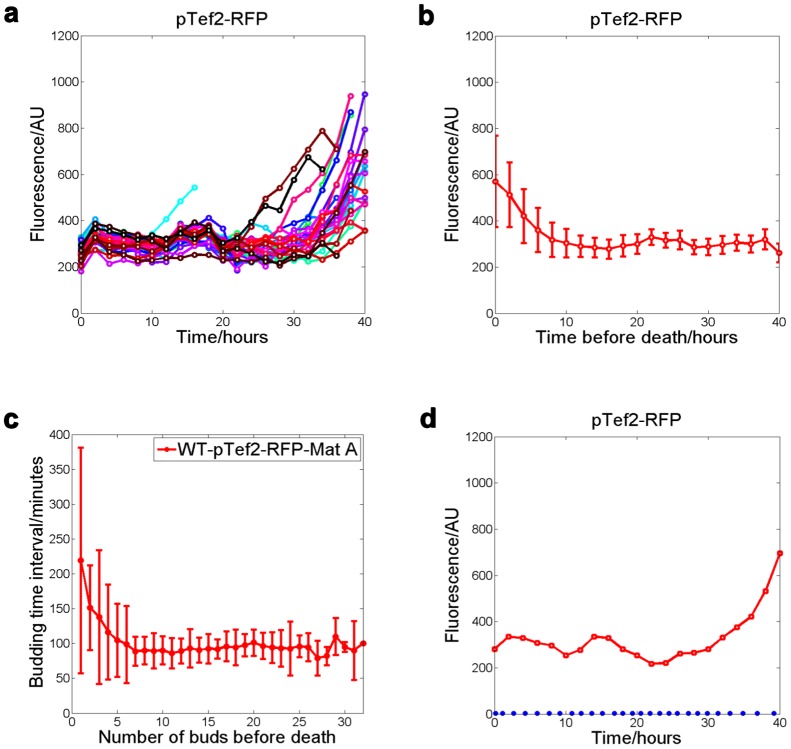
Tracking fluorescent reporter in single cells throughout their lifespan: Tef2 promoter activity increases with age. (a) fluorescent intensity of Tef2 promoter driven RFP (pTef2-RFP) is measured as a function of time in single cells. Each colored line connects data points for one single cell. RFP levels start to increase significantly after 30 hours. (b) Mean and standard deviation of the pTef2-RFP level as a function of time before death, calculated from the 30 cells in (a). pTef2-RFP level began to increase dramatically starting from 10 hours before death. (c) Expression profile of a single cell together with its budding time (blue dots). (d) Slowing down of cell cycle in the last few cell divisions. Time between two successive budding events is plotted against the number of buds before cell death. The budding time interval and its variation increase dramatically starting from 5∼6 buds before death. And pTef2-RFP level increase along with a slowing down of the cell cycle when cells get aged.

We show this application by analyzing a specific fluorescent reporter. We chose a RFP marker fused with the promoter of the *TEF2* gene: pTEF2-RFP, in the wild type *MAT-α* background. We monitored its expression in single cells as a function of their age, by taking a bright field image once every 10 minutes and a fluorescent image once every 2 hours automatically using a Nikon time-lapsed microscope. We found that the pTEF2-RFP intensity (see Methods for a description of the normalization) generally remains flat before 30 hours, and begins to increase steadily starting from 10 hours before death till the end of lifespan of the cells (Fig. 4a, b, Fig. S3a). This is surprising as TEF2 encodes the translational elongation factor EF-1 alpha; it is generally believed to be expressed constitutively. TEF2 promoter driven fluorescent reporters have been used as controls (not suppose to change) in cell culture based studies and single cell studies involving only young cells. We found that the increase of pTEF2-RFP level seems correlated with a slowing down of budding time interval, starting from 5∼6 buds before death (Fig. 4c, d). However, the increase of pTEF2-RFP is not simply caused by the slow down of cell growth, as another reporter for the heat shock factor Hsf1 in the same cells remains flat throughout the lifespan(Fig. S3b), and we tested that the Hsf1 reporter is responsive in old cells [5].

**Figure 5:**
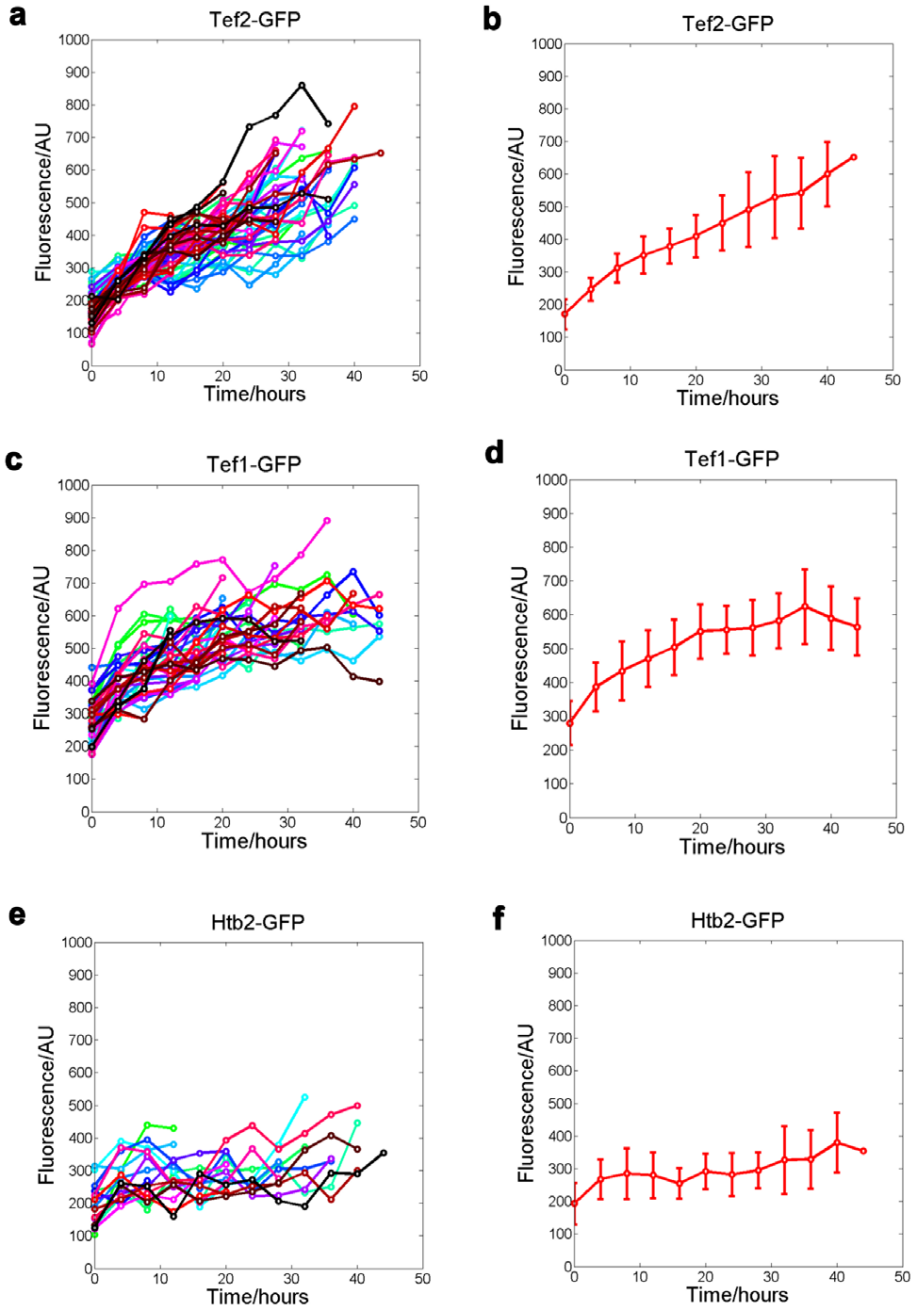
The protein level of the translational elongation factor EF1-alpha increases during aging. (a), (c), (e) fluorescent intensity of Tef2-GFP, Tef1-GFP and Htb2-GFP measured as a function of time in single cells. Each colored line connects data points for one single cell. (b), (d), (f) Mean and standard deviation of the Tef2-GFP, Tef1-GFP, and Htb2-GFP level as a function of time, calculated from the cells in (a), (c) and (e) respectively.

The increase of pTEF2-RFP level during aging could result from an increase of transcription from the TEF2 promoter, an increase in the protein synthesis of RFP, or a decrease of degradation of RFP. Since both TEF1 and TEF2 encode translational elongation factor EF-1 alpha, we wonder whether the protein level of EF-1 alpha also increases during aging, which could indicate a change of translational activity in the old cells. To investigate this, we tracked Tef1-GFP and Tef2-GFP (Tef1 and Tef2 fused with GFP at their C-termini respectively) in single mother cells throughout their lifespan using our microfluidic device. We found that Tef2-GFP intensity has about 4-fold increase on average (Fig. 5a, b) comparing old to young cells, and Tef1-GFP intensity has about 2-fold increase on average (Fig. 5c, d). In contrast, we found that for the Htb2-GFP (histone 2B fused to GFP), the trend of increase is much less obvious (Fig. 5 e, f).

We have also analyzed in the wild type strain a GFP fused to Hsp104, which encodes a heat shock protein that refold and reactivate denatured and aggregated proteins. We found that Hsp104-GFP expression has a similar trend as that of pTef2-RFP (Fig. S3c, d, e). In addition, the Hsp104-GFP level early in life showed a negative correlation to the lifespan of yeast cells (Fig. S3f). These observations are consistent with our earlier studies using the first generation microfluidic device [5].

### Age dependent asymmetry of a stress response reporter between mother and daughter cells

Budding yeast divides asymmetrically, giving rise to asymmetric segregation of age. While the mother cell progressively ages, most of its daughter cells retain the full replicative potential, independent of the age of the mother. It is assumed that this asymmetry of age is caused by asymmetric segregation of damaged materials. Indeed previous studies have shown that aggregated and oxidatively damaged proteins are preferentially retained in the mother cells [18]–[20]. However, these observations were made either in young cells or in sorted old cells at one snapshot with chemical staining. In addition, with the cell sorting, it is difficult to obtain enough truly old cells for the single cell analysis.

Here we demonstrate that we can track mother cells throughout their lifespan while monitoring the partition of molecular markers between the mother cells and their daughters, thus we can study how the asymmetry develops at molecular level as a mother ages. We chose a mKate2 reporter driven by a crippled cyc1 promoter with STRE upstream (see Methods), where STRE refers to the stress response element or binding site for the general stress response transcription factors Msn2/4 [21]–[25]. By tracking this reporter, we wish to analyze whether the age asymmetry is accompanied by the asymmetry of the general stress response reporter.

We found that 40 µm by 40 µm is the optimal size of the pensile columns for analyzing mother/daughter inheritance during the aging process, since it can trap the mother cells throughout their lifespan, at the same time allowing their daughter cells to be trapped for several cell cycles. To measure the age dependence of the partition of the reporter, we took the bright field images once every 10 minutes continuously to track the lifespan of the mother cells, and fluorescent images once every 10 minutes in 2 hours windows from 9–11 hour, 19–21 hour, and 39–41 hour after the initial cell loading, corresponding to young, middle aged, and old mothers. This protocol allows us to track the reporter during the cell division, and to minimize fluorescent damage to the cells. Since the average budding time interval of the cells is usually less than 100 minutes, 2 hours window is sufficient to cover one division process of a newborn daughter cell from a mother cell.

**Figure 6:**
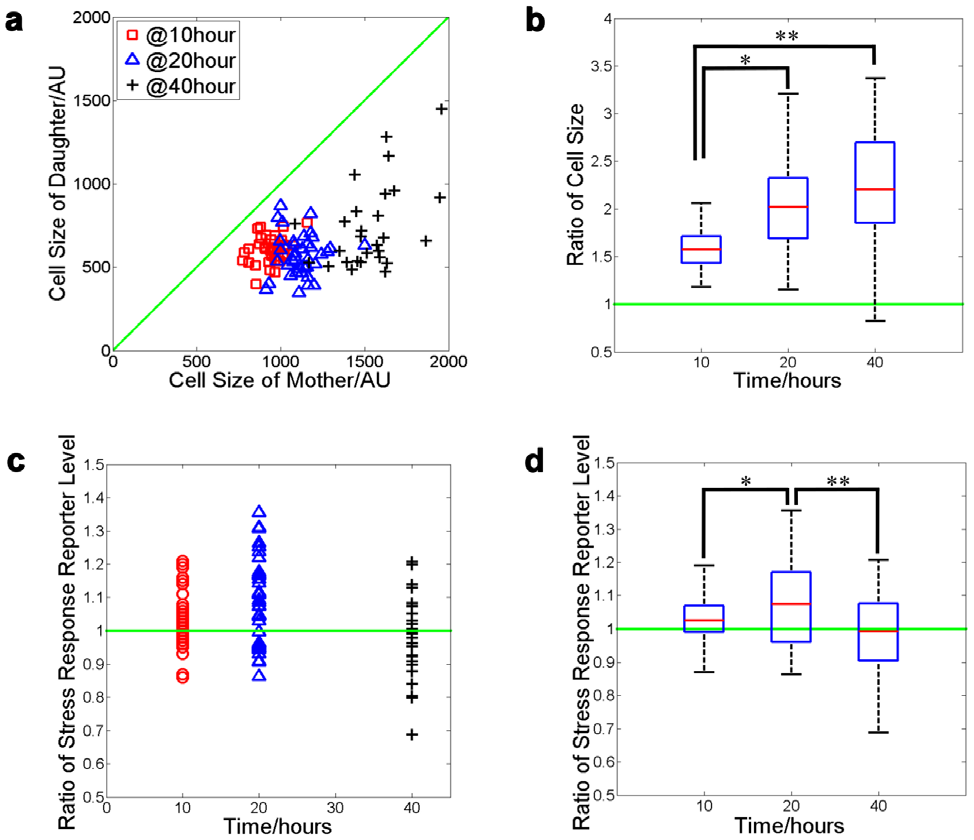
Age dependent asymmetric partitioning of a stress response reporter between mother and daughter cells. (a) The sizes of each mother–daughter pair for mother cells at different time after initial loading. As mother cell size grows with time, the size of the daughter only increase slightly. (b) box plot of the mother-daughter size ratio for the pairs measured in (a). There is a significant increase of the ratio over time (*P_T-test_ = 5.5×10^−6^, **P_T-test_ = 2.4×10^−5^). (c) The ratio of the stress response reporter level in the mother cell vs. that in its daughter. Each point represents a mother-daughter pair. Time is relative to the initial loading of the mother cells. (d) box plot of data in (c). There is a significant difference between time point 1 and 2, and point 2 and 3 (*P_T-test_ = 4.0×10^−2^, **P_T-test_ = 2.5×10^−2^).

We measured the sizes of the mother and daughter, and the normalized STRE reporter levels in the pair of the cells at the point when the division is just completed. We found that while size asymmetry increases monotonically with age (Fig. 6a, b), the asymmetry of the reporter level developed only in the middle-aged cells. The partition of the reporter is symmetric in young cells, becomes asymmetric in the middle-aged cells, and returns to symmetric in the old cells (Fig. 6c, d). One possible interpretation for this observation is that the stress response is caused by certain cellular damages that become relevant once the cells reach their middle age. While middle-aged mother cells are capable of retaining the damages to themselves, very old cells eventually lose this capability.

## Discussion

Ever since the discovery of finite lifespan in budding yeast [2], yeast aging research has been limited by the low efficiency of the replicative lifespan assay based on micro-dissection. Besides the difficulty to perform large-scale genetic screening for lifespan phenotypes, it is also unfeasible to track molecular markers during aging in single cells using the traditional method. The development of microfluidic system overcomes these difficulties and opens new directions for yeast aging study.

With the system we designed, it is possible to reach a medium throughput for lifespan assay in a semi-automated fashion. Our current device has 4 independent modules working in parallel, and it is straightforward to expand to 10 modules on a single chip. In each of the modules, around 100 mother cells can be tracked. Thus in a single experiment which typically takes 2∼3 days, the lifespan of 10 different strains can be assayed, corresponding to analyzing 1,000 mother cells and performing 30,000 micro-dissections. This would take 4∼5 skilled person 3∼4 weeks to accomplish with the traditional method. Thus the use of the microfluidic device greatly saves the time and labor needed for the lifespan analysis. Furthermore, the microfluidic setup makes it much easier to achieve constant temperature and nutritional environment, consequently reduce fluctuations from experiment to experiment.

More importantly, the system allows live tracking of molecular markers throughout the lifespan of the mother cells, making it possible to achieve a molecular description of the aging phenotype in single cells.

The new generation of microfluidic chip we presented here is a significant improvement over our previous design. Previously we used a combination of geometric confinement by the microfluidic chamber and mother cell surface labeling in conjunction with glass surface modification to retain mother cells [5]. In the new device, surface labeling is unnecessary since the mother cells are trapped by the pensile columns mechanically and jamming by the off-springs is no longer a issue. This makes the device manufacturing and cell loading much simpler, thus significantly increases the success rate of the experiment. In addition, eliminating surface labeling avoids chemical changes of the cells and minimizes the perturbation to their natural lifespan, as we showed that the lifespan of the wild type cells measured by the current microfluidic device quantitatively agrees with that from the plate assays, while the lifespan measured by the surface labeling protocol was reduced [5].

One big advantage of the new chip design is that it allows the analysis of the molecular inheritance by daughter cells from mother cells of defined age. Since cells are trapped in the gap between the pensile columns and the glass surface purely by space confinement, there is a finite probability that the daughter cell of a trapped mother cell will also be trapped. This allows us to follow the mother/daughter pair before and after the cell division. Utilizing this powerful new feature, we have analyzed the partitioning of a general stress response reporter between mother and daughter cells as a function of the age. This analysis revealed that asymmetry developed in the middle-aged mothers. However, the asymmetric effect is small (although it is statistically significant), compared to some of the previous observations (E.g, asymmetry of oxidatively damaged proteins [26]). We suspect that this may be partly due to the difference between the transcriptional activity of the promoter and the protein level. The stress level may be reflected in the activity of Msn2/4 in the nucleus and consequently the transcriptional activity of the promoter, which can be quite different between mother and daughter. However the level of the fluorescent protein mKate2 can be similar in mother and daughter immediately following cell division, due to fast diffusion of mKate2 (with a typical diffusion time of a few seconds). Thus the observed difference of the protein level can be much smaller than that of the stress level. We note that our approach can be used to analyze other molecular markers, e.g., for DNA damage and repair, mitochondrial status, protein damage and aggregation, that may be causally related to aging and asymmetrically inherited between mother and daughter cells.

Finally, the approach we developed here may be generalized to study aging in other cell culture based systems, including single cell organisms that divide symmetrically, such as the fission yeast. It has been observed that even when cells divide symmetrically, the inheritance of proteins are asymmetric and there is still age asymmetry among different lineages. With appropriately designed geometry and dimension, it is possible to trap a specific lineage (such as the lineage with the old pole) and analyze the aging phenotype by the microfluidic device.

**Note added**: While this manuscript was in preparation, we noted a paper published by Lee et al. who designed a microfluidic device for analyzing yeast aging based on a similar approach [6].

## Materials and Methods

### Microfluidic device fabrication

The mold for the chip was fabricated by the standard multilayer photolithography processes. Negative photoresists Su-8 2005, Su-8 2010 and Su-8 2025 were used to fabricate the three layers of mold on silicon wafer respectively, in order to construct the channels with pensile columns with different depths. First, the Su-8 2005 (MicroChem Corp., Newton, MA) negative photoresist was spin-coated at 5000 rpm for 30 seconds and pre-baked according to the vendor's instruction before mask aligning. Then the coated wafer was aligned and exposed (150 mJ/cm2) for 40 seconds through the mask in “direct contact” mode using a mask aligner. After exposure, the mold was post-baked and developed using Su-8 developer. Su-8 2010 photoresists was used to fabricate the second layer, spin-coated at about 3000 rpm for 40 seconds. Another mask was aligned to the Su-8 features already patterned on the coated silicon wafer, and then the wafer was exposed (150 mJ/cm2) for 60 seconds. After exposure, the mold was post-baked and developed using Su-8 developer. Su-8 2025 photoresists was used to fabricate the third layer, spin-coated at about 1500 rpm for 50 seconds. Another mask was aligned to the Su-8 features already patterned on the coated silicon wafer, and then the wafer was exposed (150 mJ/cm2) for 60 seconds. After exposure, the mold was post-baked and developed using Su-8 developer. After the mold fabrication, PDMS(parts A and B in 9∶1 ratio) was poured on the mold and allowed to cure at 70°C. Access holes to the channels were punched in PDMS and the final chip was sealed to a cover glass slide after treating with plasma.

### Yeast strains and preparation

For lifespan assay, the strains were from Brian K. Kennedy lab. WT-*MAT* ***a***: BY4741, *MAT* ***a*** his3Δ1 leu2Δ0 met15Δ0 ura3Δ0; WT-*MATα*: BY4742, *MATα* his3Δ1 leu2Δ0 lys2Δ0 ura3Δ0; sir2Δ: sir2:: KanMX in *MAT* ***a*** BY4741 background; fob1Δ: fob1::ura3 in *MATα* BY4742 background. For gene expression analysis, we constructed a strain with RFP fused to the promoter of Tef2: Δura3::pTef2-RFP-HSE-pcyc1-EmGFP-ura3 in *MATα* BY4742 background. The strains of Tef1-GFP, Tef2-GFP, Htb2-GFP and Hsp104-GFP were derived from the standard GFP strain library in WT-*MAT* ***a*** BY4741 background [27]. For aging asymmetry analysis, we constructed a strain with mKate2 [28], [29], a next generation of far-red fluorescent marker TagFP635 (mKate), fused to a crippled cyc1 promoter with STRE upstream: Δura3::STRE-pcyc1-mKate2 in *MATα* BY4742 background. Yeast cell culture were grown in YPED at 30°C overnight to OD600 1.0 and then diluted in 1: 50, and then incubated for another 6 hours at 30°C before loading into the microfluidic device by a syringe connected to an automatically controlled pump.

### Microscope and image analysis

Images were taken by a Nikon TE2000 time-lapsed microscope with 40× and 60× oil objectives. The microfluidic device was mounted on the microscope by a customized holder printed by a 3D printer. Bright field images were taken once every 10 minutes for all the experiments described in this paper. Fluorescent images were taken once every 2 hours or 4 hours in the single cell gene expression analysis. For the analysis of age dependent asymmetry of stress response reporter, fluorescent images were taken once every 10 minutes for 2 hours during 9–11 hour, 19–21 hour and 39–41 hour respectively. Customized version of cellseg 5.4, developed by Kaiyeung Lau and Zhengwei Xie (unpublished), was used to segment the bright field images of mother and daughter cells and to quantify their fluorescent signals. The fluorescent intensity is calculated by the total fluorescent signal of a cell normalized by its area. The timing of the budding events of yeast were recorded by a customized plugin of ImageJ and processed by Matlab.

### Measuring bud scar distribution of the initially loaded cells

Before loading the cells into the microfluidic device, 1 ml of yeast cells were pelleted and re-suspended in sterile PBS at OD600 1.0. They were then washed twice by PBS and re-suspended in 500 µl FITC-labeled WGA (wheat-germ-agglutinin, lectin from Triticum vulgaris; Sigma-Aldrich, UK), at a concentration of 0.5 mg/ml. Cells were gently agitated at room temperature for 20 minutes, harvested by centrifugation(13400 rpm for 1 minute) and then washed three times by PBS. Aliquots of 20 µl WGA stained cells were coated on a cover glass slide for a control analysis while the rest of it were loaded into the microfluidic device. The samples were examined by a Nikon spinning disc confocal microscope with an argon ion laser for fluorescent imaging and transmission detector for differentiation interference contrast (DIC) with z-stacks. Images were stored and reconstructed to a 3D-version by the ImageJ software.

## Supporting Information

Movie S1 **A movie of a typical experiment, made from the time-lapsed images tracking single mother cells throughout their lifespan.** The cells were trapped by the pensile columns in the microfluidic device. Bright field images were taken once every 10 minutes.
(AVI)

Figure S1 (**a**) Distribution of the number of scars for cells in an exponentially growing culture (plated on a glass slide) and those initially loaded underneath the pensile columns of different sizes. More than half of the cells initially loaded underneath the pensile columns have less than 2 bud scars. Note: the number of scars here includes the birth scar, thus the number of bud scars = the number of scars -1. (**b**) Distribution of the number of scars for cells in an exponentially growing culture and those loaded underneath the pensile columns, with data from pensile columns of different sizes combined.
(PDF)

Figure S2 (**a**) Budding time interval plotted against the number of buds before death, for the *WT-MAT* ***a*** and *sir2Δ-MAT* ***a*** strains. Cells were grouped by the number of buds before death and the average and standard deviation of the budding time interval are shown. The budding time interval and its variation increases dramatically in the last few cell divisions. (**b**) Budding time interval plotted against the number of buds before death, for the *WT-MATα* and *fob1Δ-MATα* strains. See Fig. S2a for more explanation. (**c**) The budding time interval between the 6^th^ and the 7^th^ bud of the mother cell negatively correlates with the lifespan in *WT-MAT* ***a*** strain. (correlation coefficient = −0.45, P value = 3.9×10^−3^). (**d**) The budding time interval between 7^th^ and 8^th^ bud of the mother cell negatively correlates with the lifespan in *sir2Δ-MAT* ***a*** strain. (correlation coefficient = −0.52, P value = 8.7×10^−5^). (**e**) The budding time interval between 6^th^ and 7^th^ bud of the mother cell negatively correlates with the lifespan in *WT-MATα* strain. (correlation coefficient = −0.47, P value = 1.4×10^−4^).
(PDF)

Figure S3 (**a**) The image series tracking a single mother cell. At 0 hour, 12 hour, and 24 hour, pTef2-RFP intensity of a mother cell indicated by the arrow keeps flat but is increased dramatically at 36 hour. (**b**) While pTef2-RFP intensity generally remains flat before 30 hours and begins to increase steadily starting from ∼10 hours before cell death, the intensity of HSE-GFP reporter (see Methods) remains flat from birth to death in most of the cell. The blue dots indicate the budding events of the single mother cell. (**c**) Expression level of Hsp104-GFP as a function of time in single cells. Each colored line represents the expression profile of Hsp104-GFP in one mother cell. (**d**) mean and standard deviation of Hsp104-GFP level calculated from the cells shown in Fig. S3a. (**e**) Mean and standard deviation of the budding time interval versus the number of buds before death for cells analyzed in Fig. S3a. (**f**) Hsp104-GFP level at 12 hour after initial loading negatively correlates with the lifespan of individual cells (correlation coefficient = −0.79, P value = 3.9×10^−6^).
(PDF)

Table S1 **Distribution of the number of scars for cells in an exponentially growing culture and those initially loaded underneath the pensile columns of different sizes.** Same data as in Fig. S1a & b in table format.
(PDF)
