# Supplementary material for: Single Cell Analysis of Yeast Replicative Aging Using a New Generation of Microfluidic Device
Source: PLoS One. 2012 Nov 8;7(11):e48275. doi: 10.1371/journal.pone.0048275 (PMC3493551; doi:10.1371/journal.pone.0048275)

Fig S1a Distribution of the number of scars for cells in an exponentially growing culture (plated on a glass slide) and those initially loaded underneath the pensile columns of different sizes. More than half of the cells initially loaded underneath the pensile columns have less than 2 bud scars.

Note: the number of scars here includes the birth scar, thus the number of bud scars = the number of scars -1

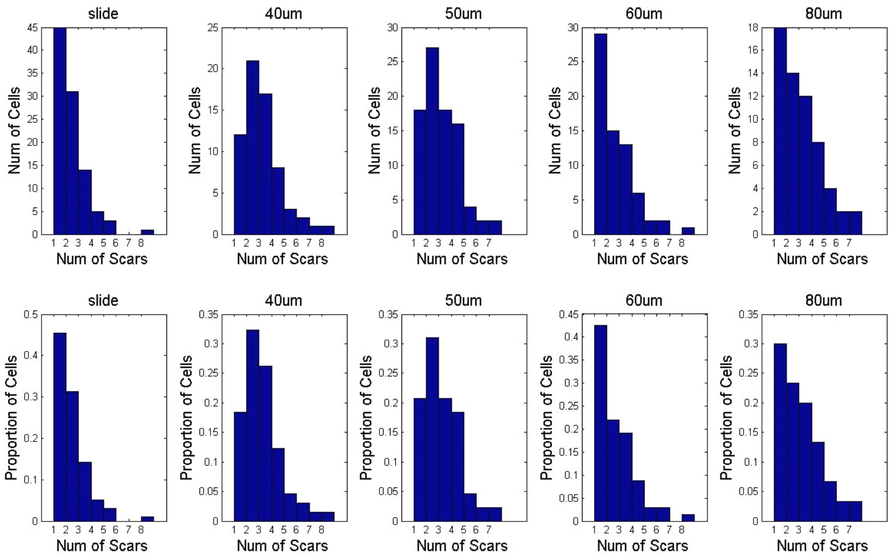

Fig S1b Distribution of the number of scars for cells in an exponentially growing culture and those loaded underneath the pensile columns, with data from pensile columns of different sizes combined.

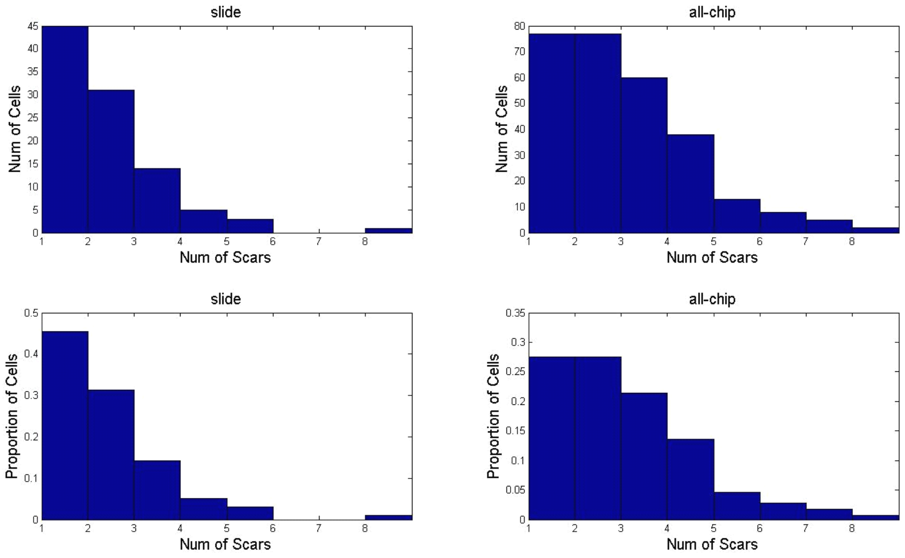

Supplement: Figure S1 — (a) Distribution of the number of scars for cells in an exponentially growing culture (plated on a glass slide) and those initially loaded underneath the pensile columns of different sizes. More than half of the cells initially loaded underneath the pensile columns have less than 2 bud scars. Note: the number of scars here includes the birth scar, thus the number of bud scars = the number of scars -1. (b) Distribution of the number of scars for cells in an exponentially growing culture and those loaded underneath the pensile columns, with data from pensile columns of different sizes combined. (PDF) [file pone.0048275.s002.pdf]
