# Supplementary material for: Single Cell Analysis of Yeast Replicative Aging Using a New Generation of Microfluidic Device
Source: PLoS One. 2012 Nov 8;7(11):e48275. doi: 10.1371/journal.pone.0048275 (PMC3493551; doi:10.1371/journal.pone.0048275)

Fig S2a Budding time interval plotted against the number of buds before death, for the *WT-MAT**a*** and *sir2Δ-MAT**a*** strains. Cells were grouped by the number of buds before death and the average and standard deviation of the budding time interval are shown. The budding time interval and its variation increases dramatically in the last few cell divisions.

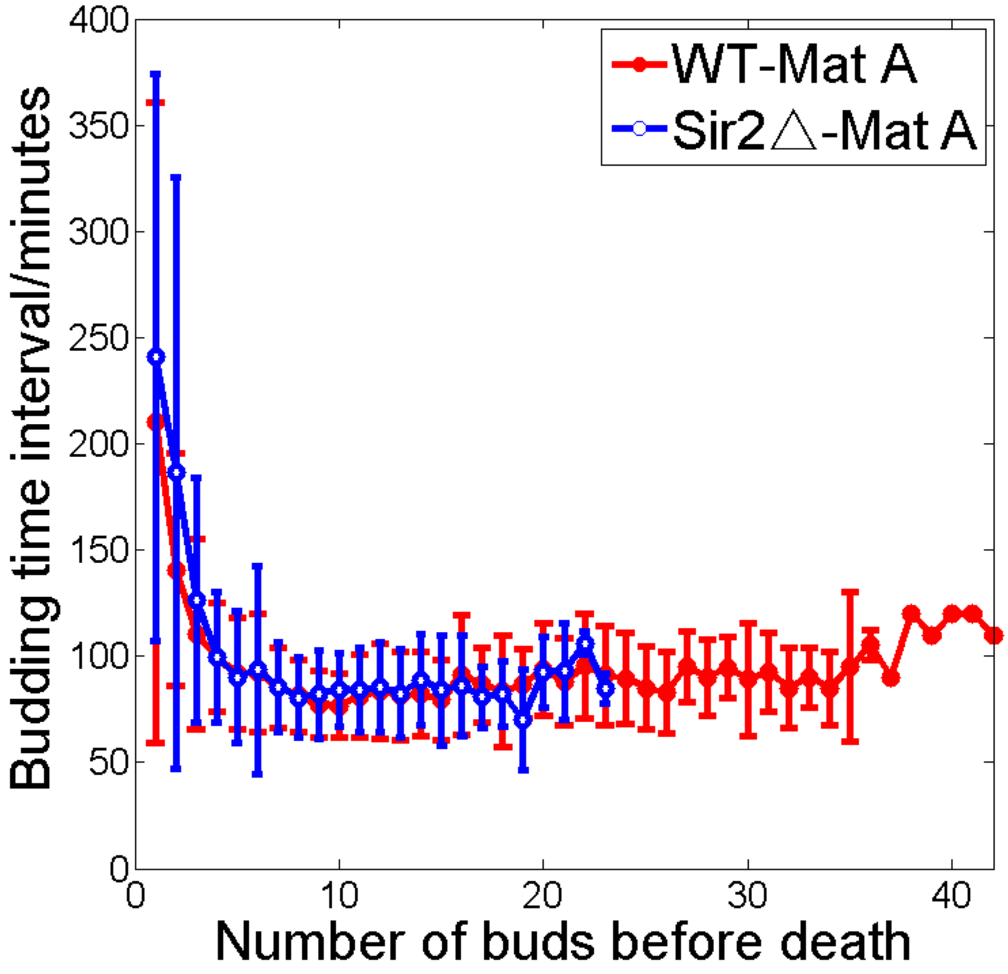

Fig S2b Budding time interval plotted against the number of buds before death, for the *WT-MATα* and *fob1Δ-MATα* strains. See Fig. S2a for more explanation.

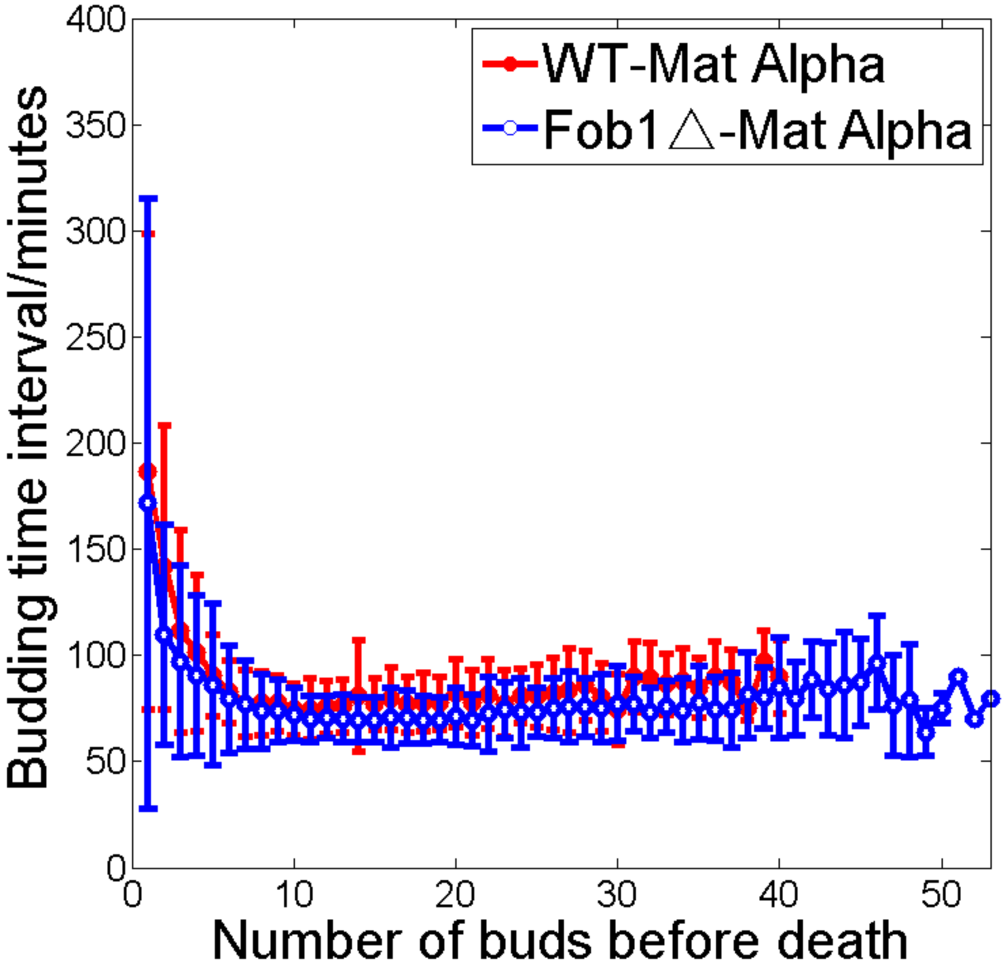

Fig S2c The budding time interval between the 6th and the 7th bud of the mother cell negatively correlates with the lifespan in *WT-MAT**a*** strain. (correlation coefficient=-0.45, P value=3.9x10^{-3}).

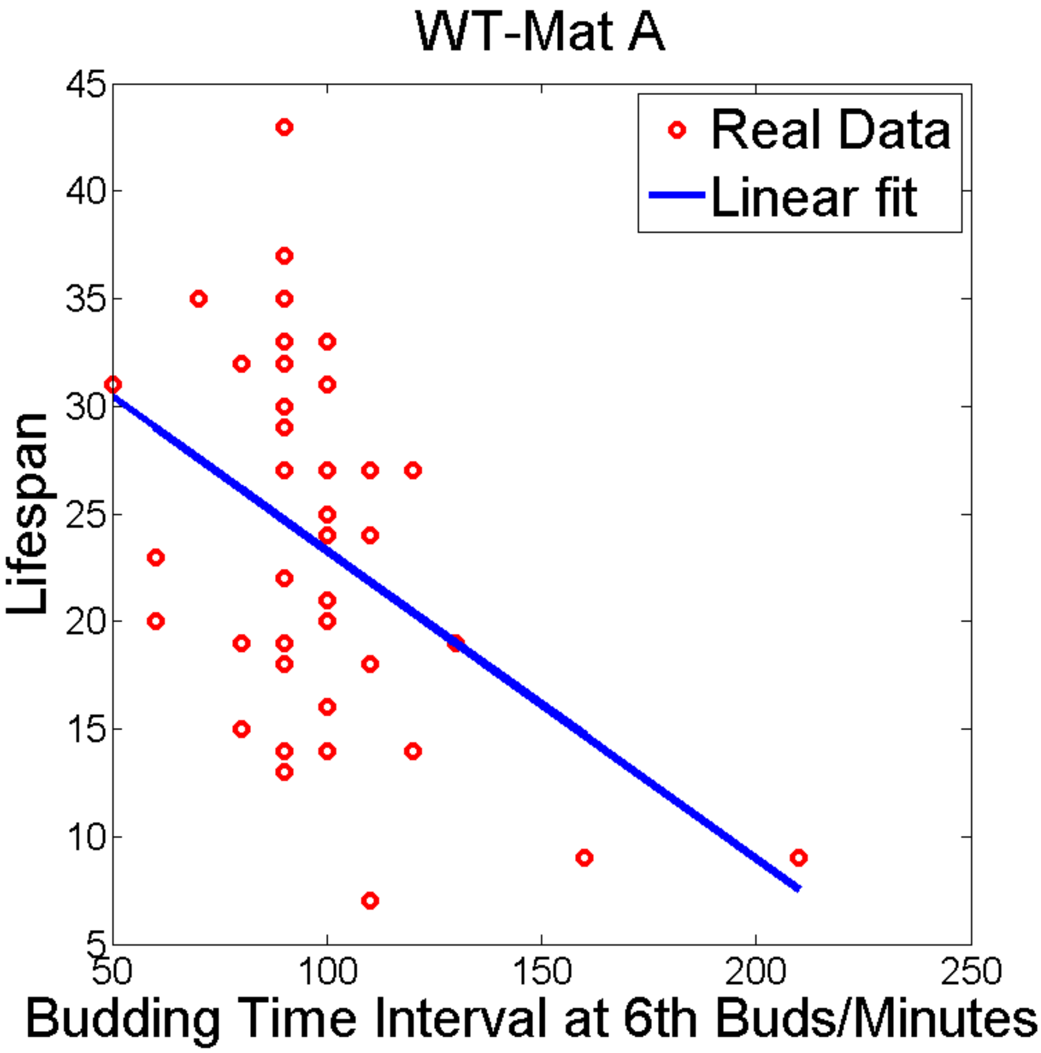

Fig S2d The budding time interval between 7th and 8th bud of the mother cell negatively correlates with the lifespan in *sir2Δ-MAT**a*** strain. (correlation coefficient=-0.52, P value=8.7x10^{-5}).

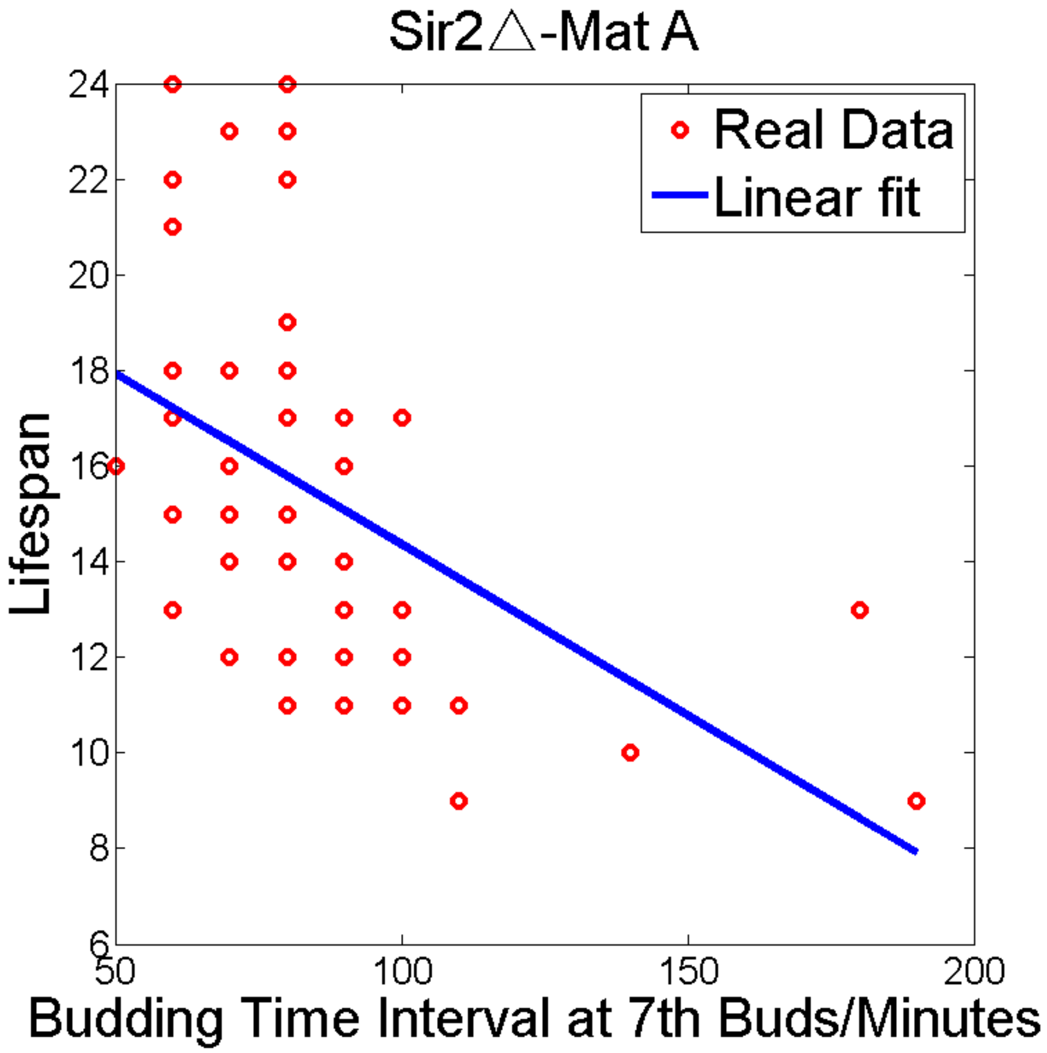

Fig S2e The budding time interval between 6th and 7th bud of the mother cell negatively correlates with the lifespan in *WT-MATα* strain. (correlation coefficient=-0.47, P value=$1.4x10^{-4}$).

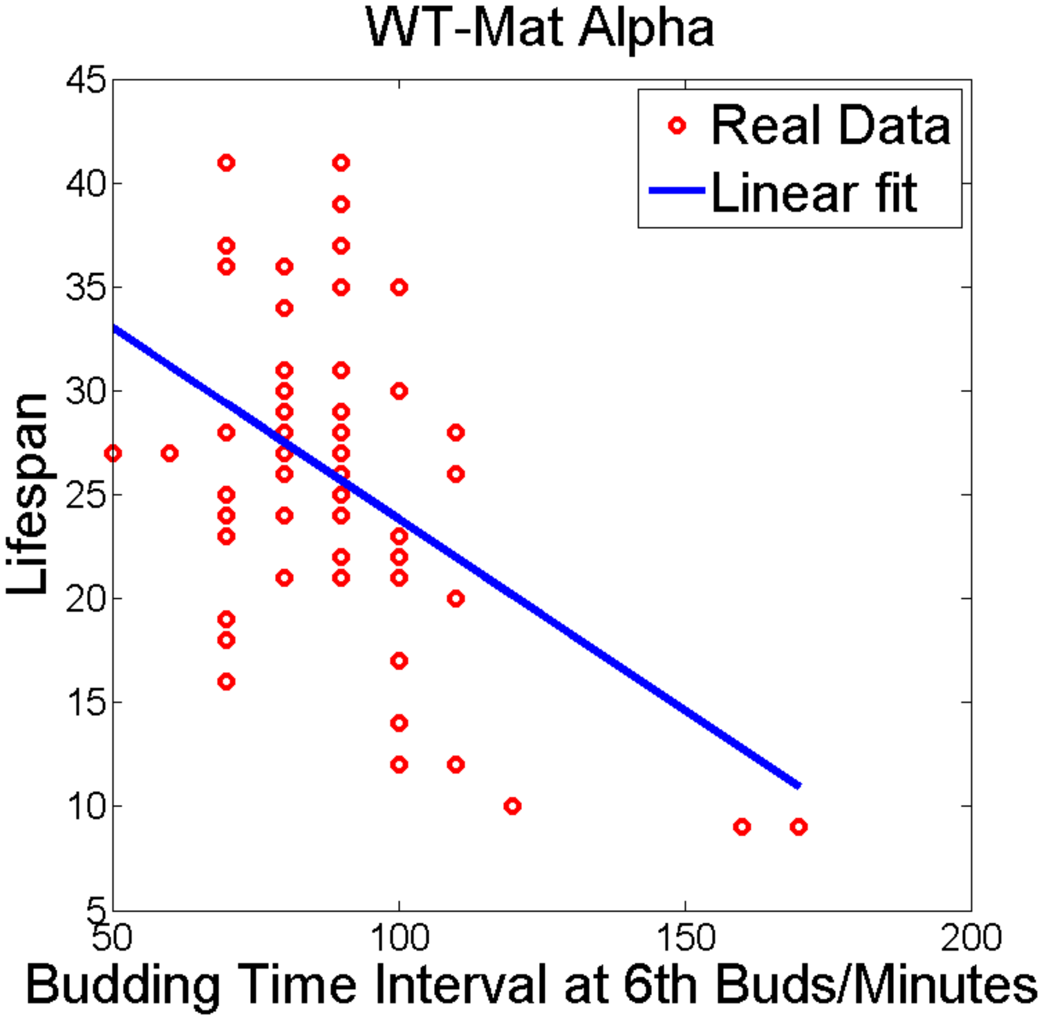

Supplement: Figure S2 — (a) Budding time interval plotted against the number of buds before death, for the WT-MAT a and sir2Δ-MAT a strains. Cells were grouped by the number of buds before death and the average and standard deviation of the budding time interval are shown. The budding time interval and its variation increases dramatically in the last few cell divisions. (b) Budding time interval plotted against the number of buds before death, for the WT-MATα and fob1Δ-MATα strains. See Fig. S2a for more explanation. (c) The budding time interval between the 6th and the 7th bud of the mother cell negatively correlates with the lifespan in WT-MAT a strain. (correlation coefficient = −0.45, P value = 3.9×10^−3^). (d) The budding time interval between 7th and 8th bud of the mother cell negatively correlates with the lifespan in sir2Δ-MAT a strain. (correlation coefficient = −0.52, P value = 8.7×10^−5^). (e) The budding time interval between 6th and 7th bud of the mother cell negatively correlates with the lifespan in WT-MATα strain. (correlation coefficient = −0.47, P value = 1.4×10^−4^). (PDF) [file pone.0048275.s003.pdf]
