# Supplementary material for: Single Cell Analysis of Yeast Replicative Aging Using a New Generation of Microfluidic Device
Source: PLoS One. 2012 Nov 8;7(11):e48275. doi: 10.1371/journal.pone.0048275 (PMC3493551; doi:10.1371/journal.pone.0048275)

Fig S3a The image series tracking a single mother cell. At 0 hour, 12 hour, and 24 hour, pTef2-RFP intensity of a mother cell indicated by the arrow keeps flat but is increased dramatically at 36 hour.

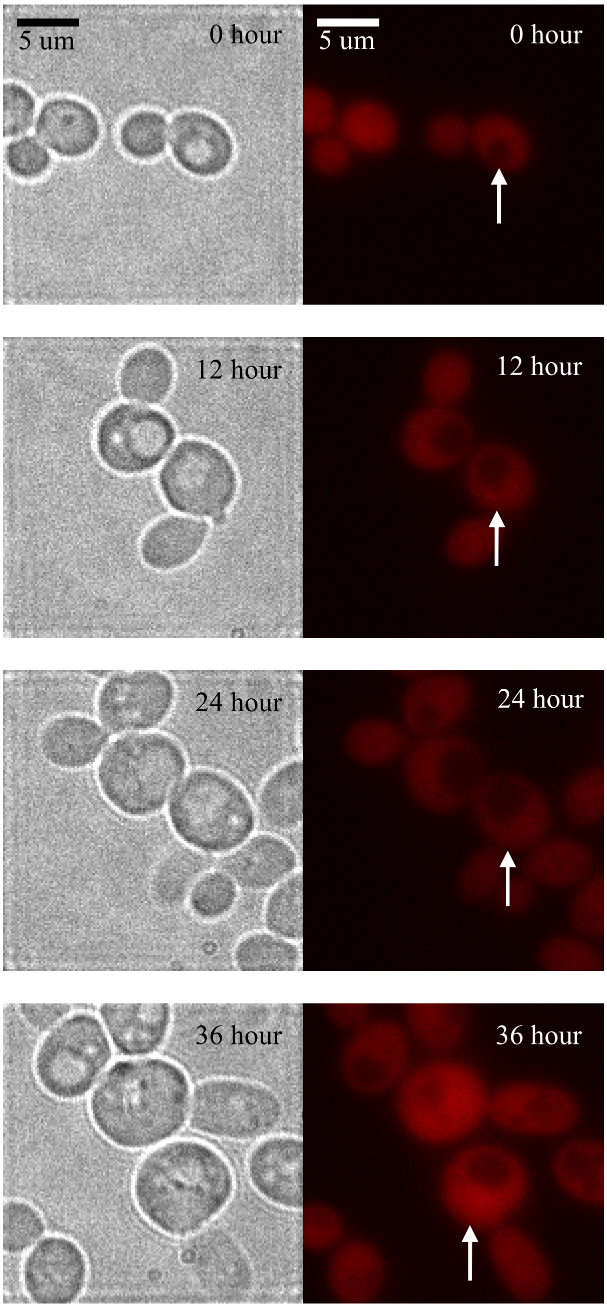

Fig S3b While pTef2-RFP intensity generally remains flat before 30 hours and begins to increase steadily starting from ~10 hours before cell death, the intensity of HSE-GFP reporter (see Methods) remains flat from birth to death in most of the cell. The blue dots indicate the budding events of the single mother cell.

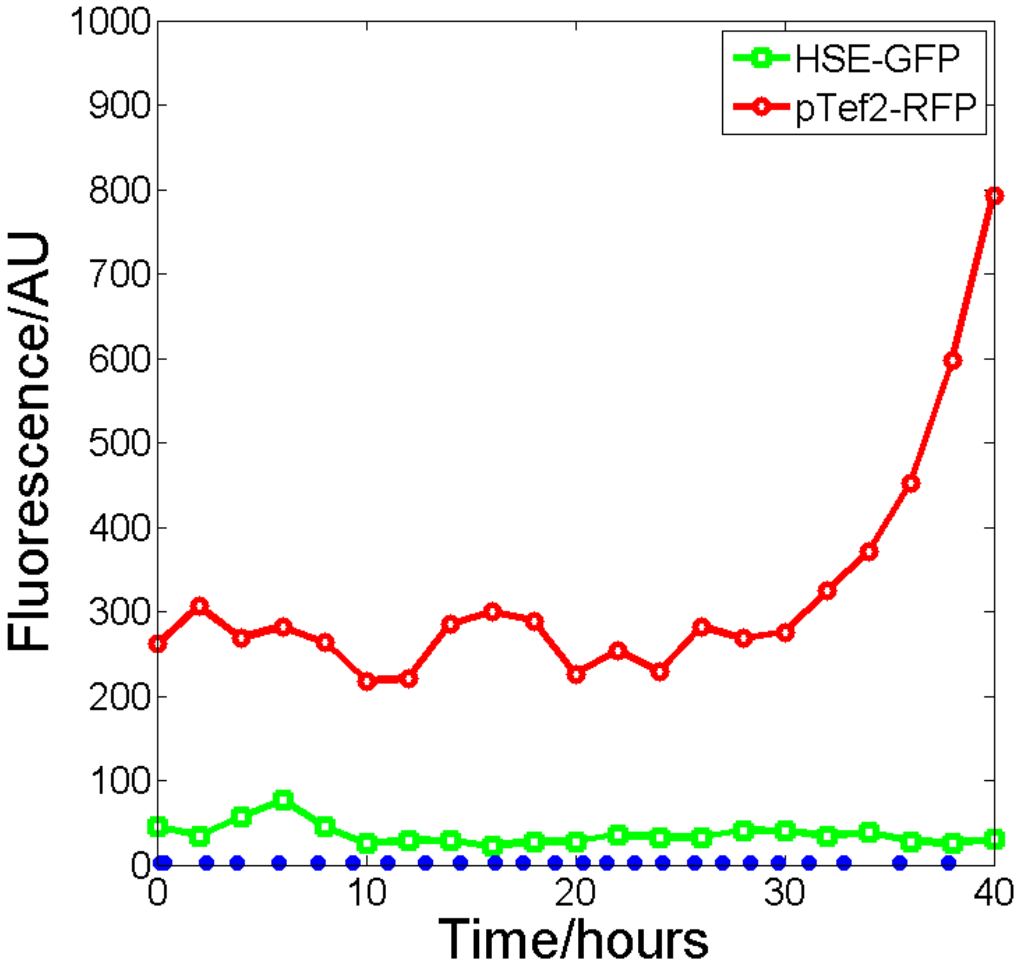

Fig S3c Expression level of Hsp104-GFP as a function of time in single cells. Each colored line represents the expression profile of Hsp104-GFP in one mother cell.

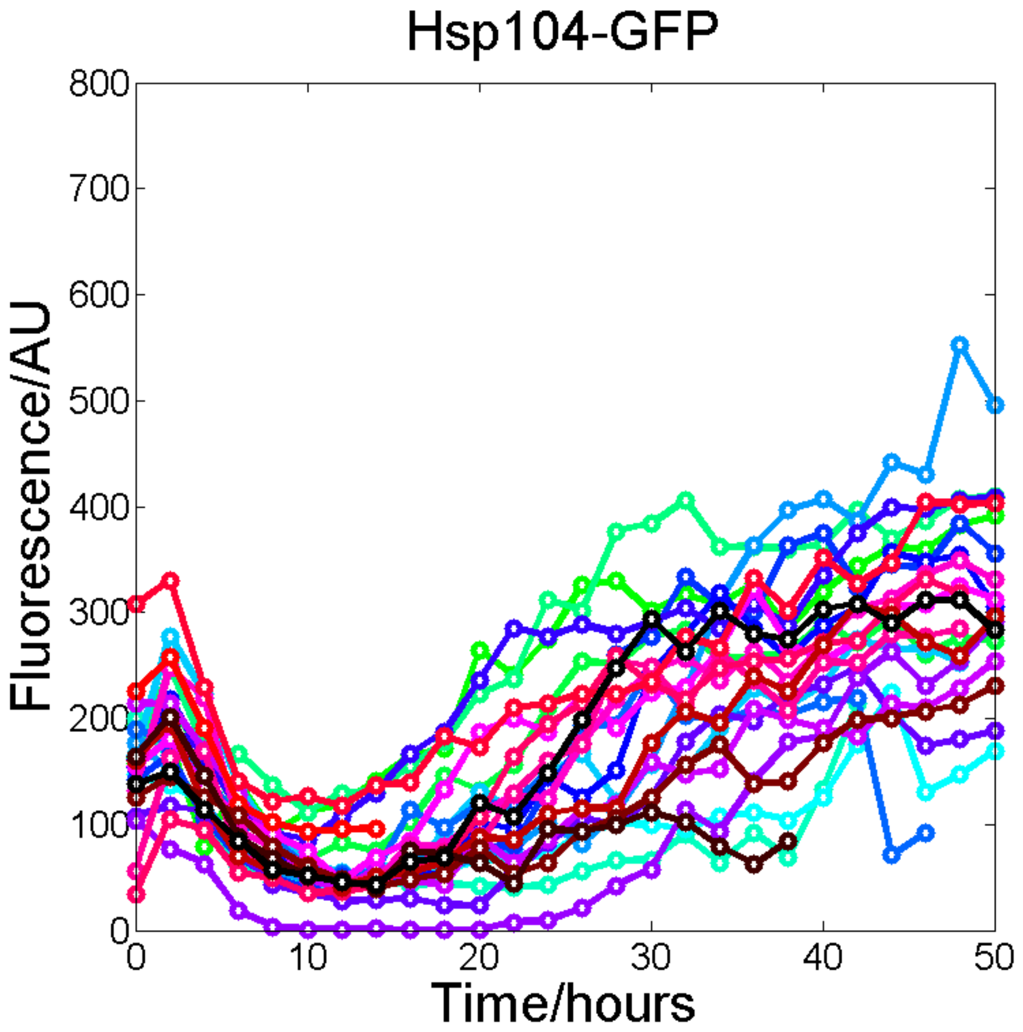

Fig S3d mean and standard deviation of Hsp104-GFP level calculated from the cells shown in Fig. S3a.

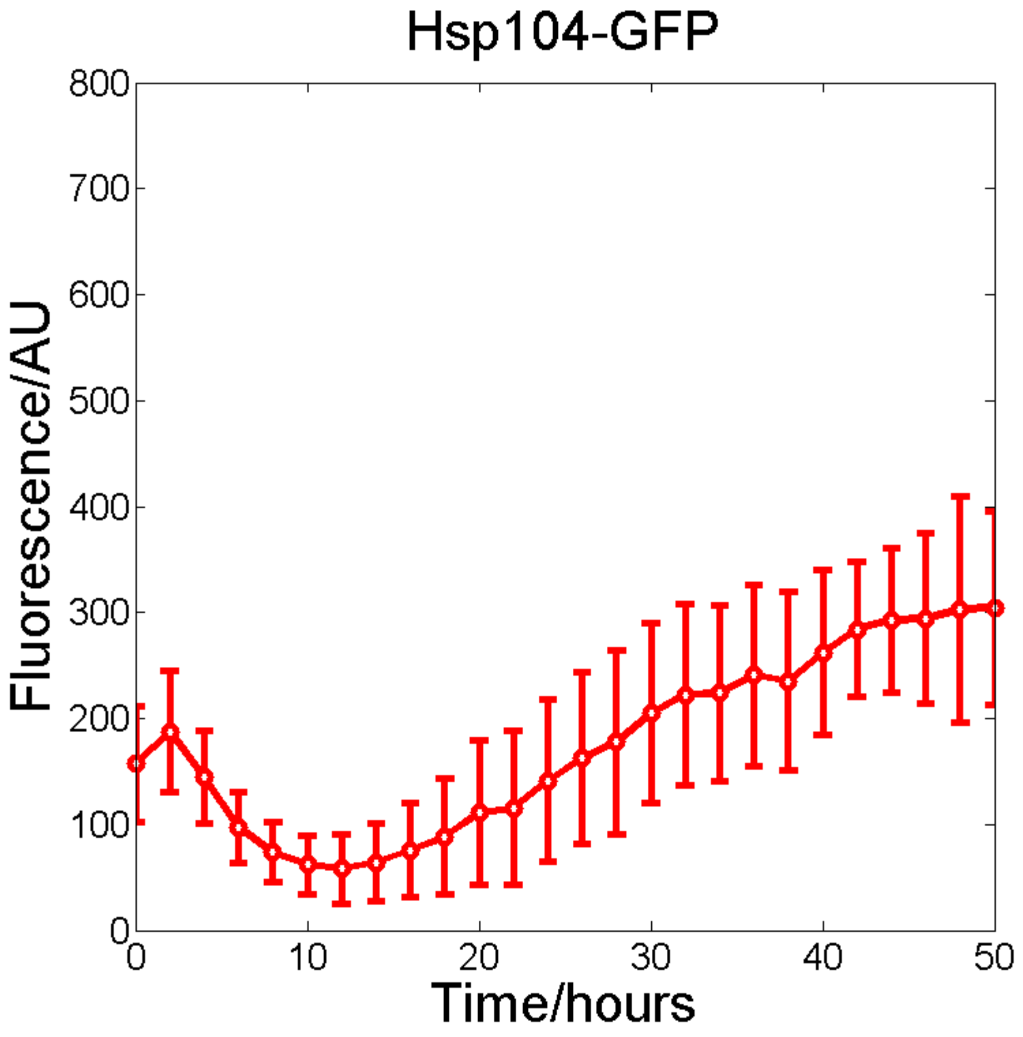

Fig S3e Mean and standard deviation of the budding time interval versus the number of buds before death for cells analyzed in Fig. S3a.

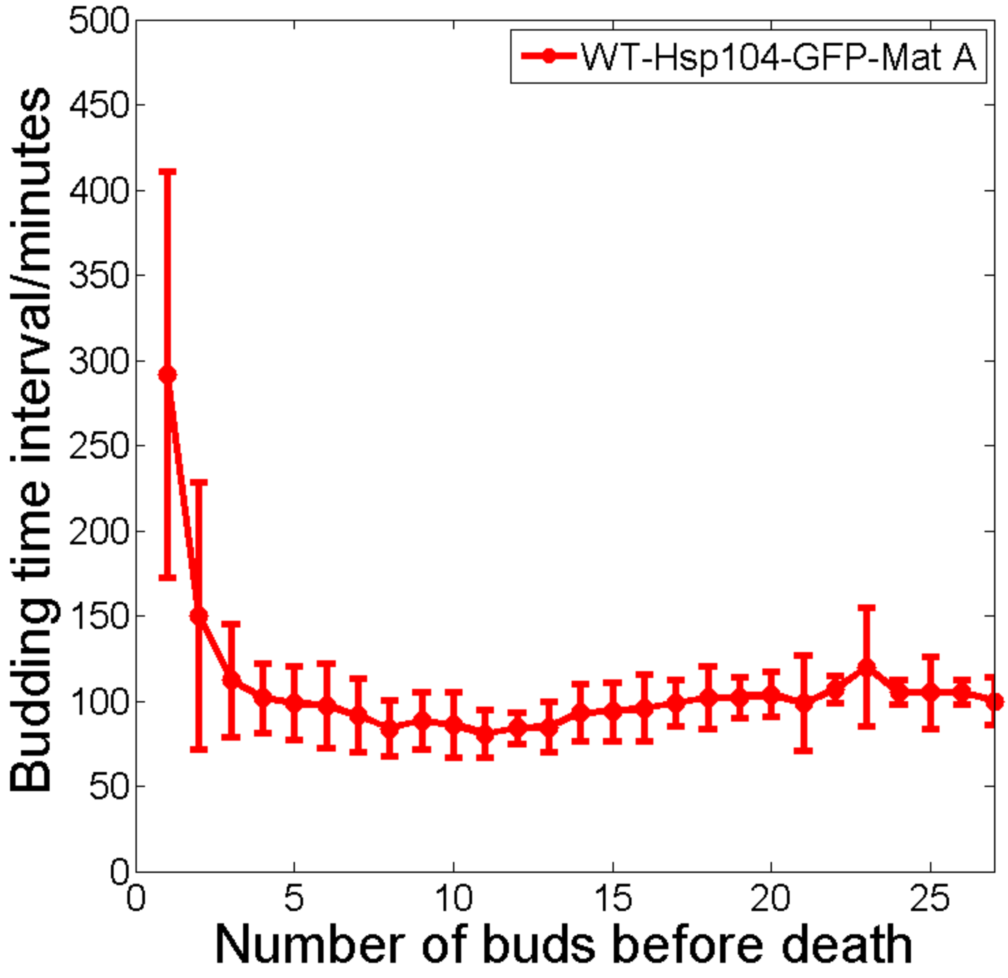

Fig S3f Hsp104-GFP level at 12 hour after initial loading negatively correlates with the lifespan of individual cells (correlation coefficient=-0.79, P value=3.9×10^{-6}).

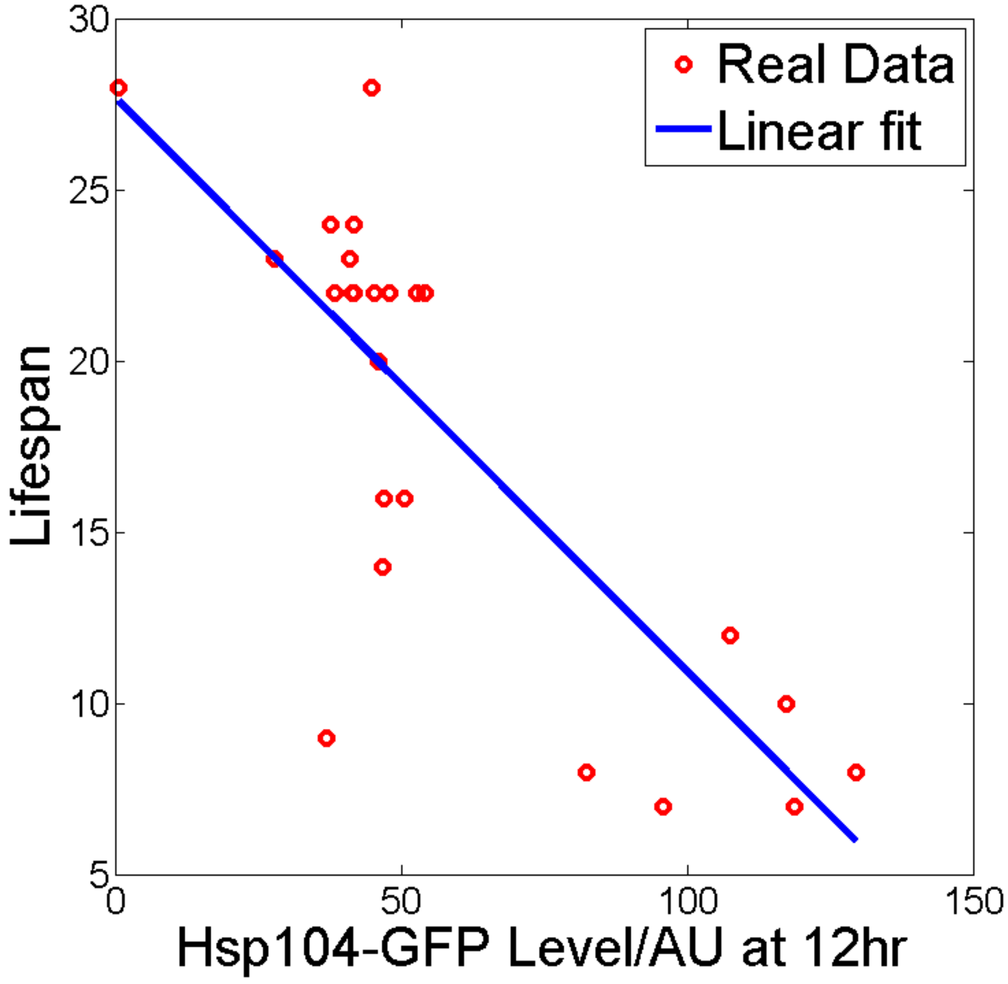

Supplement: Figure S3 — (a) The image series tracking a single mother cell. At 0 hour, 12 hour, and 24 hour, pTef2-RFP intensity of a mother cell indicated by the arrow keeps flat but is increased dramatically at 36 hour. (b) While pTef2-RFP intensity generally remains flat before 30 hours and begins to increase steadily starting from ∼10 hours before cell death, the intensity of HSE-GFP reporter (see Methods) remains flat from birth to death in most of the cell. The blue dots indicate the budding events of the single mother cell. (c) Expression level of Hsp104-GFP as a function of time in single cells. Each colored line represents the expression profile of Hsp104-GFP in one mother cell. (d) mean and standard deviation of Hsp104-GFP level calculated from the cells shown in Fig. S3a. (e) Mean and standard deviation of the budding time interval versus the number of buds before death for cells analyzed in Fig. S3a. (f) Hsp104-GFP level at 12 hour after initial loading negatively correlates with the lifespan of individual cells (correlation coefficient = −0.79, P value = 3.9×10^−6^). (PDF) [file pone.0048275.s004.pdf]
