# Supplementary material for: Single Cell Analysis of Yeast Replicative Aging Using a New Generation of Microfluidic Device
Source: PLoS One. 2012 Nov 8;7(11):e48275. doi: 10.1371/journal.pone.0048275 (PMC3493551; doi:10.1371/journal.pone.0048275)
Supplement: Table S1 — Distribution of the number of scars for cells in an exponentially growing culture and those initially loaded underneath the pensile columns of different sizes. Same data as in Fig. S1a & b in table format. (PDF) [file pone.0048275.s005.pdf]

Table S1 Distribution of the number of scars for cells in an exponentially growing culture and those initially loaded underneath the pensile columns of different sizes. Same data as in Fig S1a & b in table format.

Num of Scars	Proportion-Slide	Proportion-40um	Proportion-50um	Proportion-60um	Proportion-80um	Proportion-all chip
1	0.45	0.18	0.21	0.43	0.30	0.28
2	0.31	0.32	0.31	0.22	0.23	0.28
3	0.14	0.26	0.21	0.19	0.20	0.21
4	0.05	0.12	0.18	0.09	0.13	0.14
5	0.03	0.05	0.05	0.03	0.07	0.05
6	0.00	0.03	0.02	0.03	0.03	0.03
7	0.00	0.02	0.02	0.00	0.03	0.02
8	0.01	0.02	N/A	0.01	N/A	0.01
